# Supplementary material for: Earmarking donations to boost study participation? Evidence from a field experiment
Source: PLoS One. 2025 Sep 8;20(9):e0331498. doi: 10.1371/journal.pone.0331498 (PMC12416732; doi:10.1371/journal.pone.0331498)
Supplement: S1 Table — Participants who completed the study (n = 406) dependent to the experimental condition. (DOCX) [file pone.0331498.s001.docx]

|  | **“Earmarking”**  **(*n* = 144)** | **“Earmarking**  **with Flexibility”**  **(*n* = 128)** | **“Random”**  **(*n* = 134)** |
| --- | --- | --- | --- |
| **Age** |  |  |  |
| Mean Age | 46.3 (SD=10.4) | 48.2 (SD=11) | 46.1 (SD=10.5) |
| **Gender** |  |  |  |
| Male | 45.8% | 50.8% | 45.5% |
| Female | 49.3% | 46.9% | 54.5% |
| Other | 0.7% | 0.8% | 0 |
| Prefer not to answer | 4.2% | 1.6% | 0 |
| **Position** |  |  |  |
| Professor | 45.1% | 43.8% | 44% |
| Associate Professor | 22.9% | 25.8% | 20.1% |
| Assistant Professor | 24.3% | 21.1% | 24.6% |
| Other | 7.6% | 9.4% | 11.2% |
| **University-rank group^1^** |  |  |  |
| 1–50 | 19% | 25% | 23% |
| 51–100 | 31% | 31% | 30% |
| 101–150 | 30% | 25% | 31% |
| 151–200 | 19% | 20% | 16% |
| **Research Methods** |  |  |  |
| Quantitative | 81% | 76% | 79% |
| Qualitative | 1% | 1% | 5% |
| Both | 18% | 22% | 16% |
| **COSP^2^** |  |  |  |
| Mean COSP | 4.44 (SD=0.97) | 4.33 (SD=1.1) | 4.42 (SD=0.95) |
| *^1^Before mailing, we created four personalized survey-link batches—one for each 50-rank segment of the global top-200 list (1–50, 51–100, 101–150, 151–200). Every invitation used the link for the recipient’s group, letting us record that group—though not the exact university or rank for that matter—for every respondent.*  *^2^COSP was measured using a four-item scale. Respondents were asked to indicate the frequency with which they engage in the following activities: making all research materials available, making all data freely accessible, making all analysis methods freely available (e.g., as a pre-print), and preregistering hypotheses in advance. Responses were recorded on a five-point scale ranging from 1 (Never) to 5 (Always).* | | | |
|  | | | |
