# Supplementary material for: Earmarking donations to boost study participation? Evidence from a field experiment
Source: PLoS One. 2025 Sep 8;20(9):e0331498. doi: 10.1371/journal.pone.0331498 (PMC12416732; doi:10.1371/journal.pone.0331498)
Supplement: Attachment — Submitted filename: Response to Reviewers.docx [file pone.0331498.s003.docx]

# Response to the Editor

Thank you very much for your detailed and constructive feedback on our manuscript, and for recognizing its merit and relevance. Guided by your comments and those of the reviewers, we have undertaken a thorough revision. Below we (i) summarize the most important changes and (ii) respond point-by-point to the specific issues you raised (original comments are italicized).

*As concerns framing, it is unclear how the outcome of "study participation" contributes to the literature on earmarking, specifically its effect on the likelihood of donation. My recommendation is to frame the paper differently, starting with the puzzle of why participation in research studies remains low, and whether the promise of (earmarked) donations can boost participation. This would strike me as a more effective framing strategy and require reviewing a slightly different literature.*

Following your suggestion, the introduction now opens with the challenge of raising survey response rates and reviews the main strategies scholars currently use—most prominently: incentives. We highlight the surprising under-exploration of how to optimize donation incentives, even though they may be preferable to personal incentives in certain instances. We therefore position earmarking as a promising means of making donation incentives more effective and outline why this possibility has practical and theoretical importance. We hope you will agree that this revised framing now presents a clearer and more compelling account of our study’s contribution.

*As concerns the study design, it is unclear 1) whether academics are a relevant sample from which we can generalize*

As noted in our response to Reviewer 1, we completely agree that academics might be a population that is typically not the primary target of charitable campaigns. However, we equally see no theoretical reason to assume that academics differ systematically from the general public on traits that drive prosocial behavior. In fact, academics fit our study well precisely because— as the revised manuscript now spells out—they are relatively affluent, highly time-constrained, and, therefore, difficult to motivate with small personal incentives. Accordingly, testing donation‐based incentives in such a population should provide a meaningful test of their effectiveness.

*2) whether some outcomes used are meaningful (especially "consent" -- perhaps better labelled as "survey begun")*

Following your suggestion and the comment of Reviewer 1, we have now re-labeled the variable formerly called “*consent”* to “*survey begun”* throughout the manuscript, thereby clarifying that the variable captures the point at which a participant clicked the survey link and started the questionnaire by providing consent. All text, tables, and figures have been updated accordingly.

*and 3) if the treatment worked (manipulation checks and power calculations missing)*

We agree that we cannot be entirely sure that the manipulation worked as intended, a point that was also raised by Reviewer 2. Because the email invitation was the only vehicle for the treatment, a post-survey manipulation check would have strengthened internal validity. We explicitly acknowledge this limitation and recommend its inclusion in future work.

Sensitivity analysis: Because the sampling frame comprised all 6,711 eligible academics, N was fixed ex-ante. Instead of an a-priori power analysis, we, therefore, report a sensitivity analysis showing that, at α = .05 and 1–β = .80, the design could detect a ≥ 2-percentage-point lift (12 % vs. an illustrative 10 %).

*Moreover, it appears that the results need to be interpreted differently in that any donations for study participation, not just earmarked donations, fail to be effective. This seems like the more important finding but this baseline effect is not discussed.*

While we share your intuition given the overall low response rate, we believe that our study design does not allow for a definitive conclusion on this point, as all conditions included a $5 donation incentive. A proper test of that hypothesis would arguably require a true no-incentive control group. In the present manuscript, we, therefore, limit our conclusions to the more specific question of whether adding earmarking to a donation incentive improves participation. Our data indicate that it does not.

We again thank you for your constructive comments and guidance. We believe that our revisions have substantially strengthened the manuscript. Naturally, we are happy to make any further improvements you deem helpful.

# Response to Reviewer 1

*Reviewer #1: This article looks at the question of whether the opportunity to earmark donations affects study participation among academics at psychology departments. The motivation for this question is based on the finding in the literature that earmarking donations often affects willingness to donate, and the authors are interested in whether this extends to study participation. The manuscript is clearly written, and the experiment is cleanly executed, which make for an interesting read. However, my main comments are to do with whether study participation is a relevant outcome for donations being earmarked versus not, and whether an academic population is necessarily informative for donation behaviour more broadly. The following points expand on this and summarize a few other comments that I hope will help the authors.*

Thank you for the overall positive evaluation and the helpful suggestions that helped us to improve the manuscript.

*• First, it is not clear why the effect of earmarking donations on study participation is an interesting question because earmarking usually relates to donations to charities and international organizations, as the authors also note, whereas study participation of the sort we see in this manuscript is academic. Therefore, it is not clear why this is an interesting and relevant question and whether (academic) study participation is a relevant and useful outcome for studying the effects of earmarking donations.*

Thank you for raising this important point. In the revised manuscript, we now more clearly articulate why examining the effect of earmarking—as a specific way of designing donation incentives—on study participation is both relevant and meaningful. Specifically, we explain that donation incentives may be particularly useful in contexts involving highly affluent, time-constrained populations—such as academics—who are less responsive to small personal incentives due to high opportunity costs. In such cases, donation-based incentives offer a viable and potentially more appropriate alternative. We selected academics as our sample precisely because they exemplify such a time-poor population with high opportunity costs for whom donation incentives are particularly appropriate.

Moreover, despite their practical relevance, there is limited systematic evidence on how donation incentives can be optimized. Examining whether a well-documented design feature to increase donations—earmarking—can increase study participation thus addresses a practical need and extends the theoretical framework surrounding the earmarking construct.

• *Somewhat related to the previous point, are academics generally a good population for studying the effects of earmarking donations? In other words, academics are not usually the main target audience for charitable donation so studying their receptiveness to earmarking versus not is not necessarily informative. I realize the charity in question is relevant to academics but the literature that the manuscript is situated in is broader and the examples of charities given are also very different from the kind of society/charity used in the experiment. The conclusions drawn seem to be very broad, which is why it becomes even more important to consider whether the type of sample used in the experiment is relevant for the kinds of charities the results are alluding to.*

We acknowledge that our study examines a specific population for studying earmarking effects. However, importantly, studies on earmarking do not typically focus on a “typical donor” population either. Recent work has used general-population samples recruited via online platforms such as Prolific and MTurk (Esterzon et al., 2022; Özer et al., 2024) or nationally representative samples from 25 countries (Fuchs et al., 2020). These studies consistently find a positive effect of earmarking across varied demographic and cultural contexts, suggesting that the underlying mechanism is not specific to a particular donor group.

Moreover, while we acknowledge that academics may not be a primary target audience for large-scale fundraising campaigns, there is no theoretical or empirical reason to believe they differ systematically from the general population on dimensions relevant to prosocial behavior. In fact, evidence points in the opposite direction: higher education was found to be associated with increased charitable giving (Nakamura et al., 2025), and both empirical studies (Shaker & Palmer, 2012) and reports from institutional campaigns from U.S. universities (e.g., Anthony, 2023; WCU, 2023; UTSA, 2025) show that faculty and staff frequently engage in internal giving campaigns. These donations often support causes aligned with science, education, and equity—domains closely related to the professional identity of academic psychologists.

Most importantly, however, the primary focus of our study is not on increasing charitable donations, but on testing an intervention—earmarking—aimed at increasing survey response rates. As noted previously, we believe that academics represent a particularly difficult population to motivate for survey participation, and are therefore suited for investigating the research question at hand.

Nevertheless, we acknowledge that our sample has distinctive features that may limit the generalizability of our findings—most notably participants’ extensive experience with experimental manipulations, which can dampen the impact of such manipulations (Chandler et al., 2015; Krefeld-Schwalb et al., 2024). We now acknowledge this limitation more explicitly in the revised limitations section and call for future research to examine whether less experienced or more diverse populations respond differently, providing more generalizable insights into earmarking effects.

• *The choice of the charity makes sense given the target audience, but it is unclear whether it is one that participants would care deeply about especially in comparison to charities that are usually studied in this literature. Did the authors perhaps ask questions to gauge whether participants trusted the Society to choose the right causes, spend the money fully et cetera? Similarly, was this a charity where participants likely cared enough about it in general to care about how their donation money would be spent?*

Thank you for raising these important points. We acknowledge that our survey did not include explicit measures of participants’ trust in SIPS or the extent to which they valued its mission. While we believe many participants likely had strong confidence in SIPS’s stewardship—given its sustained engagement in the academic psychology community, its strong commitment to open and transparent research practices, and annual conferences that attract 500–1,000 attendees—we recognize that individual perceptions of trust still might vary. Likewise, concerning mission importance, we deliberately chose SIPS, a charity deeply embedded in participants’ professional community, to leverage identity congruence and value alignment, factors that have been shown to increase donation willingness (Chapman et al., 2025; Kesberg & Keller, 2021), however, we cannot be sure that mission importance was high for every participant. We now explicitly highlight this limitation in the revised manuscript and recommend that future research include direct measures of trust and mission importance, to clarify their moderating role in earmarking effects.

• *One of my main concerns when reading about the treatment conditions was whether the purposes were sufficiently different for participants to care enough to want to complete the study and be able to earmark. The authors briefly touch upon this in the conclusion, but this may be a more significant factor than presented because, in the real world, charities, especially international ones, are donating to very disparate causes with a lot of differentiation. For instance, when donating to something like UNICEF, you can often choose between different causes and different countries and, therefore, it is much more likely that those who are donating will care about which cause their donation goes to. In comparison, roughly the same set of recipients between three purposes that are all to do with helping academics within the field of psychology is unlikely to evoke the same reaction or interest in differentiating between the various causes. It may also, in general, not be a set of causes that participants have strong feelings about in any case, again especially in comparison with the types of recipients that large charities, whether national or international, help.*

We appreciate the thoughtful observation that the three SIPS purposes offered by the organization—supporting an open-access preprint platform and two forms of travel grants—are much more closely related than the highly differentiated options donors typically encounter at large humanitarian NGOs. We fully agree that this limited cause differentiation is a genuine limitation of our design when it comes to assessing the external validity of our findings. At the same time, that very homogeneity provides a useful feature: because the choices are not very heterogeneous, any increase we observe can likely only stem from the perceived impact pathway of earmarking, and not from strong pre-existing cause preferences. In other words, our study offers a conservative test of the mechanism. If earmarking produces a measurable uplift even when the available options are considerably similar, it should work at least as well—and plausibly better—when donors can choose among more differentiated causes. To make this rationale explicit, we have expanded the Discussion to describe our design as a conservative test and to call for future research that systematically varies cause differentiation as a potential moderator of the earmarking effect.

• *In the analysis, it is unclear why "consent" is a dependent variable of interest. Participants who consented may or may not have reached the treatment page so it’s not obvious what information can be gleaned from this, and therefore it’s a bit odd that this is a main dependent variable in the analysis.*

Thank you for highlighting the point with the “Consent” variable. We noticed that we provoked this issue in how we labeled and described this outcome variable. We recognize that this label may have been misleading, and we appreciate the opportunity to clarify both the variable’s role and how it fits within our experimental design.

In our study, treatment exposure occurred in the invitation email, which participants received before deciding whether to click through to the survey. As such, participants who chose not to enter the survey had already been exposed to the treatment condition. Therefore, the variable we initially called “consent” does not represent pre-treatment baseline behavior or a decision uninfluenced by condition. What we were capturing with that variable was whether participants clicked on the survey link and began the survey (indicated consent on the first page of the study). As suggested by the editor, a more accurate and informative label for this outcome is “survey begun.” We have revised the manuscript accordingly, updating all references to the variable across the text, tables, and figures to reflect this clarification.

• *The authors summarize their power calculation, but it would be helpful to know whether a change of 2 percentage points would be meaningful in this context, as it seems rather low. It would also be helpful to know what the basis is for the 10% assumed participation rate, unless that is simply an example in which case stating that would add clarity.*

We appreciate the request for additional detail and clarify the two points below.

To set a plausible baseline participation rate for our sensitivity analysis, we reviewed studies involving the same population—academic psychologists—who were asked to complete studies on a similar topic. Two studies stood out as particularly relevant: Donnelly (2019) and Anderson et al. (2022). Donnelly’s paper does not report how many individuals were contacted, so no participation rate can be determined. In contrast, Anderson et al. (2022) report a 9 % participation rate among psychology and management faculty worldwide. Based on this figure—and our own informal experience with similar, time-pressured academic samples—we used 10 % as a rounded, illustrative baseline. This avoids false precision and errs slightly on the conservative side (since detecting a 2-point lift is more difficult on a higher baseline). We now explicitly clarify in the manuscript that the 10 % figure is evidence-informed but illustrative.

Regarding the practical significance of a 2-percentage-point increase: in our sample of 6711 potential participants, a lift from 10 % to 12 % in the two earmarking conditions (*N* = 4474) would correspond to 90 additional donations. At a per-participant donation of $5, this would yield $450 in additional funds—nontrivial for a nonprofit like SIPS. Furthermore, a 2-point increase on a 10 % baseline represents a 20 % relative improvement, a meaningful gain in most real-world donation contexts. From a researcher’s perspective, we also view a 2 % increase in participation—achieved through a simple, cost-free design feature—as a worthwhile outcome. Finally, we emphasize that we performed a **sensitivity analysis,** not a power analysis to determine what effect size would be detectable with our given sample size.

We again thank you for your constructive comments, which, we believe have helped to further strengthen our paper.

References:

Anthony, B. (2023, June 27). Employee giving participation rises for both faculty and staff. *UC Davis News*. <https://www.ucdavis.edu/news/employee-giving-participation-rises-both-faculty-and-staff>

Anderson, C. G., McQuaid, R. W., & Wood, A. M. (2022). The effect of journal metrics on academic resume assessment. *Studies in Higher Education, 47*(11), 2310–2322. <https://doi.org/10.1080/03075079.2022.2061446>

Chandler, J., Paolacci, G., Peer, E., Mueller, P., & Ratliff, K. A. (2015). Using Nonnaive Participants Can Reduce Effect Sizes. *Psychological Science*, *26*(7), 1131–1139. <https://doi.org/10.1177/0956797615585115>

Chapman, C. M., Spence, J. L., Hornsey, M. J., & Dixon, L. (2025). Social identification and charitable giving: A systematic review and meta-analysis. *Nonprofit and Voluntary Sector Quarterly*. Advance online publication. <https://doi.org/10.1177/08997640251317403>

Donnelly, K., McKenzie, C. R. M., & Müller-Trede, J. (2019). Do publications in low-impact journals help or hurt a CV? *Journal of Experimental Psychology: Applied, 25*(4), 744–752. <https://doi.org/10.1037/xap0000228>

Esterzon, E., Lemmens, A., & Van den Bergh, B. (2023). Enhancing Donor Agency to Improve Charitable Giving: Strategies and Heterogeneity. *Journal of Marketing*, *87*(4), 636–655. <https://doi.org/10.1177/00222429221148969>

Fuchs, C., de Jong, M. G., & Schreier, M. (2020). Earmarking Donations to Charity: Cross-cultural Evidence on Its Appeal to Donors Across 25 Countries. *Management Science*, *66*(10), 4820–4842. <https://doi.org/10.1287/mnsc.2019.3397>

Kesberg, R., & Keller, J. (2021). Donating to the ‘right’ cause: Compatibility of personal values and mission statements of philanthropic organizations fosters prosocial behavior. *Personality and Individual Differences, 168*, 110313. <https://doi.org/10.1016/j.paid.2020.110313>

Krefeld-Schwalb, A., Sugerman, E. R., & Johnson, E. J. (2024). Exposing omitted moderators: Explaining why effect sizes differ in the social sciences. Proceedings of the National Academy of Sciences - PNAS, 121(12), e2306281121. <https://doi.org/10.1073/pnas.2306281121>

Nakamura, J. S., Woodberry, R. D., Weziak-Bialowolska, D., Kubzansky, L. D., Shiba, K., Padgett, R. N., Johnson, B. R., & VanderWeele, T. J. (2025). Childhood predictors of charitable giving and helping across 22 countries in the Global Flourishing Study. *Scientific Reports, 15*(1), Article 14493. <https://doi.org/10.1038/s41598-024-77950-1>

Özer, Ö., Urrea, G., & Villa, S. (2024). To Earmark or to Nonearmark? The Role of Control, Transparency, and Warm-Glow. *Manufacturing & Service Operations Management*, *26*(2), 739–757. https://doi.org/10.1287/msom.2022.0096

Shaker, G. G., & Palmer, M. M. (2012). 6: The Donors Next Door; Raising Funds From Faculty for Faculty Development Centers. *To Improve the Academy*, *31*(1), 85–99. <https://doi.org/10.1002/j.2334-4822.2012.tb00675.x>

University of Texas at San Antonio (UTSA). (2025). *Faculty & staff giving campaign*. UTSA Giving. <https://www.utsa.edu/giving/how-to-give/faculty-staff-giving.html>

Western Carolina University (WCU). (2023, November 15). *Faculty, staff giving campaign results in participation rate, total donors increase*. WCU Stories. <https://www.wcu.edu/stories/posts/faculty-staff-giving-campaign-results-in-participation-rate-total-donors-increase.aspx>

# Response to Reviewer 2

*Reviewer #2: Introduction
The introduction advances the earmarking concept in charitable giving and provokes the primary question of the study: whether earmarking would boost participation in research studies with donation-based incentives. It defines the gap in generalizing donation behavior findings to study participation and introduces the experimental design.
• Sudden shift to the "dark side" of earmarking. The transition can be facilitated by a bridging sentence that logically links the good and bad sides of earmarking.*

Thank you for this helpful comment. We agree that the transition to the potential downsides of earmarking was abrupt in the earlier version. In our updated manuscript, we have completely revised the introduction and now present the rationale for integrating the Earmarking with Flexibility condition when talking about the experimental conditions. Specifically, we added a bridging sentence to better transition from a primarily donor-oriented analysis of earmarking to one that considers the perspective and practical constraints of charitable organizations. With this revised structure and the new introductory sentence, we hope you will agree that the rationale for integrating and testing the flexible earmarking condition is now presented in a more coherent and natural way.

*• Limited theoretical foundation connecting donation motivation and study participation. Draw on self-determination theory or prosocial behavior spillover literature to account for why earmarking effects would generalize to participation.*

Thank you for this valuable suggestion. We agree that the theoretical connection required clarification and have significantly strengthened it in the revised manuscript. We now integrate **Leverage-Saliency Theory of Survey Participation** (Groves et al., 2000) with **Self-Determination Theory** (SDT), specifically its **Cognitive Evaluation Theory** (CET) subcomponent (Deci & Ryan, 1985; Ryan & Deci, 2000), to establish a cohesive framework.

As articulated in Leverage-Saliency Theory of Survey Participation, participation decisions reflect a cost-benefit tradeoff where study attributes gain influence on the decision to participate through their leverage (weight in decision-making) and salience (psychological prominence). We position donation incentives as a positively leveraged attribute by offering psychological benefits aligned with **impure altruism** (Andreoni, 1990).

Critically, earmarking amplifies these benefits by directly satisfying two CET-based psychological needs:

1. **Autonomy**: Granting choice over where donations flow (Patall et al., 2008)
2. **Competence**: Enabling perceived impact through project-specific allocation (Aknin et al., 2013)

As we elaborate further in the manuscript, the fulfillment of these two basic needs offers compelling grounds to assume that earmarking enhances intrinsic motivation—not only for donating but also for engaging in behaviors such as study participation.

*Literature Review
Literature review documents prior earmarking studies, psychological accounts (control, effect, transparency) and effect of earmarking on donation behavior.
Surface-level analysis of mechanisms (impact, control, transparency). Expand this section to distinguish between these mechanisms in depth and examine which might not be effective in a study participation context.*

Thank you for your suggestion—it helped us clarify our thinking. In the revised manuscript, we now explain more clearly how earmarking works by linking it to CET, which suggests that people are more motivated when they feel a sense of control and competence. We also point out that earmarking can increase transparency, which in turn may build trust. In addition, we suggest that psychological ownership may be an important difference between donations made with one’s own money and donations funded by someone else. When people don’t feel that the money is truly theirs, they may feel less emotionally involved, less personally responsible for the impact, and less influenced by trust—especially in low-risk situations. This may help explain why earmarking might be less effective in donation incentives compared to situations where people donate their own money.

*• Failure to report charity-based rewards in survey research and theories of research participation motivation. Integrate literature on incentives of donation in research and motivational aspects in responding to surveys.*

We appreciate your suggestion to better integrate the literature on donation-based incentives and the motivational foundations of survey participation. In the revised manuscript, we now explicitly reference existing work on the effectiveness of both personal and donation incentives in research contexts—highlighting the relative lack of systematic research on the latter. Additionally, we connect the use of incentives to the Leverage–Saliency Theory of Survey Participation, which provides a useful framework for understanding how and when such incentives influence participation decisions.

*• The critique lacks a strong concluding sentence that summarizes the gap and clearly conveys the need for this research.*

Thank you for pointing this out. We understand the comment to refer to the final paragraph of the introduction. We have substantially revised this section to more clearly highlight the research gap and our study’s contributions. The new ending emphasizes that this is the first experimental test of earmarking within study incentivization, extends the earmarking literature beyond traditional donation settings, and introduces a novel, more flexible format aligned with constraints faced by charity organizations. We hope that you agree that this revised ending now more effectively communicates the relevance and significance of our research.

*Methodology
The study uses a large-scale field experiment with 6,711 academic researchers randomly assigned to three conditions: Random, Earmarking, and Earmarking with Flexibility. The main dependent measures are completion and consent rates.
• Uncertainty regarding randomization procedure (manual or computerized, stratified by variables like university?). Detail randomization procedure and include balance checks on age, gender, rank.*

In total, we ordered the 6,711 eligible scientists by their university’s position in the global ranking and then manually assigned them in ascending order, in repeating fashion, to one of three experimental conditions. Of the 406 scientists who finished the survey, the groups are virtually identical on the characteristics we recorded in the survey (Please see the updated S 1 Table below):

|  | **“Earmarking”**  **(*n* = 144)** | **“Earmarking**  **with Flexibility”**  **(*n* = 128)** | **“Random”**  **(*n* = 134)** |
| --- | --- | --- | --- |
| **Age** |  |  |  |
| Mean Age | 46.3 (SD=10.4) | 48.2 (SD=11) | 46.1 (SD=10.5) |
| **Gender** |  |  |  |
| Male | 45.8% | 50.8% | 45.5% |
| Female | 49.3% | 46.9% | 54.5% |
| Other | 0.7% | 0.8% | 0 |
| Prefer not to answer | 4.2% | 1.6% | 0 |
| **Position** |  |  |  |
| Professor | 45.1% | 43.8% | 44% |
| Associate Professor | 22.9% | 25.8% | 20.1% |
| Assistant Professor | 24.3% | 21.1% | 24.6% |
| Other | 7.6% | 9.4% | 11.2% |
| **University-rank group^1^** |  |  |  |
| 1–50 | 19% | 25% | 23% |
| 51–100 | 31% | 31% | 30% |
| 101–150 | 30% | 25% | 31% |
| 151–200 | 19% | 20% | 16% |
| **Research Methods** |  |  |  |
| Quantitative | 81% | 76% | 79% |
| Qualitative | 1% | 1% | 5% |
| Both | 18% | 22% | 16% |
| **COSP^2^** |  |  |  |
| Mean COSP | 4.44 (SD=0.97) | 4.33 (SD=1.1) | 4.42 (SD=0.95) |
| *^1^Before mailing, we created four personalized survey-link batches—one for each 50-rank segment of the global top-200 list (1–50, 51–100, 101–150, 151–200). Every invitation used the link for the recipient’s group, letting us record that group—though not the exact university or rank for that matter—for every respondent.*  *^2^COSP was measured using a four-item scale. Respondents were asked to indicate the frequency with which they engage in the following activities: making all research materials available, making all data freely accessible, making all analysis methods freely available (e.g., as a pre-print), and preregistering hypotheses in advance. Responses were recorded on a five-point scale ranging from 1 (Never) to 5 (Always).* | | | |
|  | | | |

Differences across conditions are small (≤ 2 years in age, ≤ 5 percentage points in gender composition, and very similar distributions in academic rank and university-rank group, as well as in research methods the academics work with), which suggests that randomization successfully produced demographically comparable participant groups.

• *No manipulation check for whether or not participants appreciated and saw the earmarking opportunity. Future experiments must include a post-task assessment of manipulation.*

We agree that assessing participants’ awareness of the earmarking opportunity would strengthen the interpretation of our findings. Therefore, future experiments on this topic should indeed include a manipulation check to ensure that participants notice and understand the earmarking condition. This point also aligns with the Leverage–Saliency Theory of Survey Participation, which holds that the influence of any survey attribute depends not only on its personal relevance (leverage) but also on whether it is sufficiently highlighted or brought to the respondent’s attention (salience) during the participation decision. We now acknowledge this limitation explicitly in the manuscript.

*• Equivalence in donation targets erodes the salience of the choice. Future experiments must employ more dissimilar donation opportunities.*

We fully agree that this limited cause differentiation is a limitation of our design when it comes to assessing the external validity of our findings. At the same time, this limited differentiation provides a useful feature: because the choices are not very dissimilar, any increase we observe can likely only stem from the perceived impact pathway of earmarking, not from strong pre-existing cause preferences. In other words, our study offers a conservative test of the mechanism. If earmarking produces a measurable uplift even when the available options are considerably similar, it should work at least as well—and plausibly better—when donors can choose among more differentiated causes. To make this rationale explicit, we have expanded the Discussion to describe our design as a conservative test and to call for future research to systematically vary cause differentiation as a potential moderator of the earmarking effect.

• *No attrition analysis (who withdrew after consent). Report the drop-off rate and assess if attrition by condition differed.*

Thank you for pointing this out. Attrition was comparable across conditions. Specifically, 28 out of 162 participants failed to finish in the Random condition (17.3%), 32 out of 176 in the Earmarking condition (18.2%), and 32 out of 160 in the Earmarking with Flexibility condition (20%).

Exploratory two-tailed two-proportion *z*-tests showed no significant differences:

- Random vs. Earmarking: *z* = −0.22, *p* = .826
- Random vs. Earmarking with Flexibility: *z* = −0.63, *p* = .529
- Earmarking vs. Earmarking with Flexibility: *z* = −0.42, *p* = .674

Pooling the two earmarking conditions (64 / 340 = 19.0%) and comparing that aggregate attrition rate with the Random condition likewise revealed no significant difference, *z* = 0.42, *p* = .674.

*• Findings show no significant material effect of earmarking on completion or consent rates. Equivalence testing suggests any effect is smaller than the pre-specified 2% cut-off. Click rate exploratory analysis suggests a small, non-significant difference.*

*Discussion
The discussion interprets null findings, identifies limitations, and specifies implications for the use of earmarking in research volunteer incentives.*

*• Disjointed limitations section; no flow and connections to theory. Reorganize into a single paragraph with each limitation connected to its effect on results and proposing future research directions.*

We agree that the previous Limitations section read like a list. We have now reorganized it into a single cohesive paragraph framed explicitly by Leverage–Saliency Theory of Survey Participation. Each limitation is now tied to leverage ($5 amount, academic sample, trust, mission importance, limited cause differentiation) or salience (experience with experimental manipulations, subtle cue visibility) and paired with a concrete future research step (e.g., testing larger amounts, more diverse samples, greater cause differentiation, manipulation checks).

*• Limited discussion of the theoretical explanations for why earmarking may not prompt participation. Incorporate behavioral economic theories or motivation theories describing the distinction between giving and participating.*

We thank you for this comment and now address it in two places. First, as noted earlier, in the expanded section on earmarking, we highlight psychological ownership as a potential key moderator that distinguishes giving one’s own money from allocating external funds through participation. This distinction helps explain why earmarking may lose motivational pull when implemented in a donation incentive aimed at increasing participation, compared to a context where it is used to increase giving. Second, in the Discussion, we outline a concrete follow-up study: experimentally increasing psychological ownership—for example, through a choice-based incentive (Stoffel et al., 2024)—to test whether heightened ownership amplifies the impact of earmarking.

*• The shift to applied implications is abrupt. Add a bridging sentence connecting the theoretical findings to practical recommendations.*

Thank you for highlighting this issue. We revised the discussion in two ways to address your comment. First, the Implications section now follows immediately after the presentation of results, while Limitations and Future Research appear later. This reordering removes the sudden jump into practical recommendations, you noted. Second, we inserted an explicit bridge that now opens the Implications subsection and links our empirical findings to the ensuing practical guidance:

“While future research is needed to better understand and contextualize the absence of effects observed in this study, practical implications can already be drawn from our findings.”

We hope this revision clarifies the connection between our findings and their practical implications and improves the overall coherence of the discussion.

We thank you for your many helpful comments and suggestions. We believe your feedback helped us to further improve our paper.

References:

Aknin, L. B., Dunn, E. W., Whillans, A. V., Grant, A. M., & Norton, M. I. (2013). Making a difference matters: Impact unlocks the emotional benefits of prosocial spending. *Journal of Economic Behavior & Organization*, *88*, 90–95. <https://doi.org/10.1016/j.jebo.2013.01.008>

Andreoni, J. (1990). Impure Altruism and Donations to Public Goods: A Theory of Warm-Glow Giving. *The Economic Journal (London)*, *100*(401), 464–477. https://doi.org/10.2307/2234133

Deci, E. L., & Ryan, R. M. (1985). *Intrinsic motivation and self-determination in human behavior*. Plenum Press.

Groves, R. M., Singer, E., & Corning, A. (2000). Leverage-Saliency Theory of Survey Participation: Description and an Illustration. *Public Opinion Quarterly*, *64*(3), 299–308. <https://doi.org/10.1086/317990>

Patall, E. A., Cooper, H., & Robinson, J. C. (2008). The effects of choice on intrinsic motivation and related outcomes: A meta-analysis of research findings. Psychological Bulletin, 134(2), 270–300. [https://doi.org/10.1037/0033-2909.134.2.270](https://psycnet.apa.org/doi/10.1037/0033-2909.134.2.270)

Ryan, R. M., & Deci, E. L. (2000). Self-Determination Theory and the Facilitation of Intrinsic Motivation, Social Development, and Well-Being. *The American Psychologist*, *55*(1), 68–78. <https://doi.org/10.1037/0003-066X.55.1.68>

Stoffel, S. T., Chaki, B., & Vlaev, I. (2024). Testing a decoy donation incentive to improve online survey participation: Evidence from a field experiment. *PloS One*, *19*(2), e0299711–e0299711. https://doi.org/10.1371/journal.pone.0299711
