# Supplementary material for: Earmarking donations to boost study participation? Evidence from a field experiment
Source: PLoS One. 2025 Sep 8;20(9):e0331498. doi: 10.1371/journal.pone.0331498 (PMC12416732; doi:10.1371/journal.pone.0331498)
Supplement: Attachment — Submitted filename: Response_to_Reviewers_auresp_2.docx [file pone.0331498.s004.docx]

# Response to the Editor

*Thank you for submitting your manuscript to PLOS ONE. We have reached a decision of "minor revision" (without external review). R1 is satisfied with the revisions as they address their points raised. R2 recommends a minor revision, asking you to do a better job in interpreting the substantive effects of the treatment and to discuss the wider implications of whether earmarking is overall a good strategy to boost donations, especially given a host of literature on the negative performance effects of earmarking.*

Thank you for the positive feedback and for giving us the opportunity to revise the manuscript and address the remaining points raised by Reviewer 3. Guided by your comments and those of the reviewer, we have undertaken a further revision. Below we respond point-by-point to the specific issues that have been raised (original comments are italicized).

*We agree with R2's points, even though we are also aware that some of these asks fall out of the scope of your analysis. Please do a good faith effort to address these points. We would recommend you take a look at these studies on the effectiveness of earmarking, which should help contextualize the findings.*

*https://doi.org/10.1086/736339
https://doi.org/10.1111/rego.12632
https://doi.org/10.1017/S0020818323000085*

*Please note: the revised version will not be sent back to the reviewers. We hope this will accelerate the decision-making process.*

Thank you for providing these references. Combined with Reviewer 3’s request to consider the potential harms of earmarking, this prompted us to examine the issue more closely and to qualify our recommendation regarding its use. In the previous version, we had already acknowledged that earmarking may be suboptimal for charities because it reduces their flexibility in allocating funds. We now also highlight evidence from Heinzel et al. (2023) and Heinzel et al. (2025a, b), showing that earmarking can be associated with worse project performance in the context of international development organizations. Accordingly, in the implications section we now state that earmarking should only be implemented when its potential advantages clearly outweigh these downsides.

# Response to Reviewer #3

*This article presents findings from a survey experiment on whether allowing participants to earmark charitable contributions given as an incentive for participation increases the response rate. Counter to expectations derived from literature on charitable giving, it finds that it does not. The article is clearly written and makes a succinct point. I think it is worthy of publication. However, I would suggest some further revisions to clarify the findings as well as their contribution and implications.*

*Most importantly, while the brief literature review covers key explanations for why earmarking should encourage charitable giving, it does so in a theoretical register. To help interpret the findings, the authors should provide substantive discussion of the findings of this literature, including the type of effects and their magnitude. For example, the article states that earmarking encourages charitable giving. However, it would be helpful to clarify whether it incentivises people to give who otherwise would not, or if it increases the amount they give. Relatedly, the authors should provide evidence on the magnitude of the effect for charitable giving found in previous studies to provide a sense of what effects we might be looking for in this experiment. Importantly, there must be some minimum level of effectiveness of earmarking for the research to be relevant, so that should be clearly stated. (This is the most important mechanism to provide substantive insights on, but it would be helpful to also provide brief substantive discussion of the other mechanisms highlighted in the literature review).*

We appreciate your positive feedback on our manuscript. We also thank you for highlighting the importance of specifying the nature and magnitude of earmarking effects in prior research. We have revised the manuscript to address these points more explicitly:

First, we have now more clearly specified the nature of earmarking effects in charitable giving. In the revised manuscript, we now explicitly specify that, while theoretically earmarking could increase both the willingness to donate and the amount donated, empirical findings more consistently show effects on willingness to donate. We argue that it is precisely this increased willingness to donate rather than increases in donation amounts that positions earmarking as a potentially valuable intervention in the context of incentivizing survey participation.

Second, regarding your suggestion to discuss the magnitude of earmarking effects from prior studies, we agree that setting clear expectations is important. However, we are cautious about making direct comparisons because previous research has examined different outcomes (e.g., webpage engagement in Costello & Malkoc, 2022; willingness to donate in Fuchs et al., 2020). Since our study is the first to test the effect of earmarking on study participation, we considered it more appropriate to base expectations on effect sizes from studies that directly target this outcome—thereby avoiding an apples-to-oranges comparison between willingness to donate and willingness to participate in a study.

In this regard, meta-analyses of web- and electronic-based survey research report that study participation can increase substantially—by odds ratios ranging from 1.39 to 2.43—when financial incentives are offered (David & Ware, 2014; van Gelder et al., 2018; Edwards et al., 2023). Assuming a baseline participation rate of 10%, these odds ratios translate into increases of roughly 4 to 12 percentage points, illustrating that participation can respond substantially to changes in incentive structures. Based on this, we set a 2 percentage-point increase (from a 10% baseline) as the minimum effect size that would render earmarking practically worthwhile in our context. From the researcher’s perspective, an effect of this magnitude could potentially justify the additional organizational effort required to implement earmarked donations, including establishing a collaboration with a charity, ensuring proper fund allocation, and managing follow-up communication. From the charity’s perspective, the expected increase in donations could potentially outweigh the downsides of earmarking. This rationale is now explicitly reflected in the revised manuscript.

*As a second point, the article claims that there is no evidence of harms of earmarking, and it might yield benefits. I was sceptical of this claim for two reasons: (1) As I understand it, earmarking is bad for charities, as it prevents them from using funds in the most effective way; and (2) presumably there is some additional cost to the researchers, even if marginal, to manage this data. The claims here should be clarified accordingly*

We appreciate this point and agree that the potential downsides of earmarking—such as reduced flexibility for charities and additional administrative burdens for researchers—are important to acknowledge. Our original statement that earmarking “does not harm” referred specifically to participation rates. We have now made this explicit and qualified our recommendation to use earmarking by noting that its implementation should always be weighed against potential downsides identified in prior research (e.g., reduced flexibility, worse project performance) and the added administrative costs for researchers, and should therefore be implemented only when its potential advantages clearly outweigh these downsides.

*Third, I wondered if the authors might expand a bit more on what they think explains the lack of results. This may be easier to clarify once the expectations are more clearly stated (by drawing out the substantive content in the literature review as noted in point 1)*

As we now make clear, thanks to your earlier suggestion, our expectation was that earmarking would motivate more people to participate. One possible reason it did not do so in this context is a difference in psychological ownership of the donated funds between donations made with one’s own money and those made through externally funded donation incentives. While we had implied this previously, we now make it more explicit in the future research section:

“A promising direction for future studies is to examine the role of psychological ownership in differentiating donations made with one’s own money from externally funded donation incentives, as theorized before, which could be a reason why earmarking in this particular instance did not prove effective.”

*Minor points:
- The response rates of this study and other studies of 9-10% are first mentioned in the analysis strategy. I wondered if this could be mentioned earlier to further motivate the study.*

We appreciate the suggestion. In our view, the reference to response rates from other studies serves primarily to provide a plausible baseline for the sensitivity analysis, rather than to motivate the study itself. For this reason, we believe it fits most naturally within the analysis strategy section. If you or the editor still believe this information should appear earlier, we would be happy to try to integrate it accordingly.

*- The first sentence of the abstract: “Charitable donations are often the best way to incentivise study participation” – requires citation and/or evidence, or should be toned down.*

We thank you for this comment and have revised the first sentence of the abstract to state more cautiously that, in many cases, charitable donations can be the most suitable form of incentivization. This is supported by reasoning presented later in the manuscript, where we note that budgetary, ethical, or participant-specific constraints may at times preclude the use of personal incentives.

*- It would be helpful to clarify throughout that this is about increasing response rates to surveys.*

Following your suggestions, we have revisited the manuscript to ensure that our focus on survey response rates is consistently clear. While the abstract and introduction already made this focus explicit, we have further reinforced it by adding the phrase “in attracting respondents for surveys” when describing prior research and by specifying “to increase response rates” when discussing the importance of understanding how to implement donation incentives optimally. We hope that these adjustments make the objective of the paper clearer from the outset.

*- The article states that “affluent or time-poor individuals may face opportunity costs that no realistic cash payment within the study budget can offset [15, 16]. Under such circumstances, even modest personal incentives are unlikely to attract these participants.” ◊ but then presumably donation-based incentives also wouldn’t impact them? If there is evidence that people would be more persuaded by a donation than direct payment, stating it more explicitly would be important. Otherwise, I think the justification can rest on the idea that sometimes it is not appropriate to pay respondents and that it can be difficult (especially with data protection/management requirements, and for online surveys where you would not be able to hand cash directly to respondents)*

Thank you for this comment and for prompting this clarification. Indeed, the findings by Khan et al. (2020) show that when the amount that can be paid per person is small, donation incentives can be more motivating than direct payments. While both incentive types may have limited cognitive valuation due to the low amount, donations add an affective component that can make them more compelling. We now make this mechanism explicit in the manuscript.

*- The article mentions the importance of trust to earmarking – but I wondered if it is also possible that if people really trust the organisation, they don’t care about earmarking because they figure the organisation can decide what to do with the resources more effectively than they can.*

Thank you for this thoughtful observation. We already note in the manuscript that trust is likely of lower importance in the context of donation incentives for study participation, given the relatively small amounts involved and the lower psychological ownership of the donated funds. This suggests that the trust-based mechanism through which earmarking can increase donations may be weaker in this setting. As you point out, it is also possible that when trust in the organization is high, offering earmarking may not increase donations via this pathway because participants believe the organization can allocate resources more effectively than they can. However, even when trust does not play a role in a particular instance, this does not necessarily mean that earmarking would have no positive effect. Arguably, the main reason for its motivational potential lies not in increasing trust, but in its ability to satisfy the basic psychological needs for autonomy and competence, as explained by cognitive evaluation theory.

*- On the ethics statement, clarify the language - The study was not anonymous, rather no identifying data was collected/respondents remained anonymous*

Thank you for pointing this out. We have removed the wording that described the study as “anonymous,” as the manuscript already specifies that no identifying data were collected.

*- I agree with one of the previous reviewers’ comments that a brief reflection on the possibility that the $5 donation had no effect on participation at all is worth mentioning – even though of course the study did not test this, pointing it out as a pathway for future study may be valuable.*

Again thank you for this comment. It prompted us to adapt the first limitation referring to the low amount. While we already note that the $5 incentive may have been too small, we now also connect this with the specific characteristics of donation incentives identified earlier, namely, lower psychological ownership of the funds. It may be the case that, due to this lower psychological ownership, earmarking within a donation incentive only has a measurable effect at higher amounts (compared to the charitable donation scenario).

References:

Costello, J. P., & Malkoc, S. A. (2022). Why Are Donors More Generous with Time Than Money? The Role of Perceived Control over Donations on Charitable Giving. *The Journal of Consumer Research*, *49*(4), 678–696. <https://doi.org/10.1093/jcr/ucac011>

David, M. C., & Ware, R. S. (2014). Meta-analysis of randomized controlled trials supports the use of incentives for inducing response to electronic health surveys. Journal of Clinical Epidemiology, 67(11), 1210–1221. <https://doi.org/10.1016/j.jclinepi.2014.08.001>

Edwards, P. J., Roberts, I., Clarke, M. J., DiGuiseppi, C., Woolf, B., & Perkins, C. (2023). Methods to increase response to postal and electronic questionnaires. Cochrane Database of Systematic Reviews, 2023(11), MR000008.

<https://doi.org/10.1002/14651858.MR000008.pub5>

Fuchs, C., de Jong, M. G., & Schreier, M. (2020). Earmarking Donations to Charity: Cross-cultural Evidence on Its Appeal to Donors Across 25 Countries. *Management Science*, *66*(10), 4820–4842. <https://doi.org/10.1287/mnsc.2019.3397>

Heinzel, M., Cormier, B., & Reinsberg, B. (2023). Earmarked Funding and the Control–Performance Trade-Off in International Development Organizations. *International Organization*, *77*(2), 475–495. https://doi.org/10.1017/S0020818323000085

Heinzel, M., Reinsberg, B., & Zaccaria, G. (2025a). Core funding and the performance of international organizations: Evidence from UNDP projects. *Regulation & Governance*, *19*(3), 957–976. <https://doi.org/10.1111/rego.12632>

Heinzel, M., Reinsberg, B., & Siauwijaya, C. (2025b). Understanding Resourcing Trade-offs in International Organizations: Evidence from an Elite Survey Experiment. *The Journal of Politics*. https://doi.org/10.1086/736339

Khan, U., Goldsmith, K., & Dhar, R. (2020). When Does Altruism Trump Self-Interest? The Moderating Role of Affect in Extrinsic Incentives. *Journal of the Association for Consumer Research*, *5*(1), 44–55. <https://doi.org/10.1086/706512>

van Gelder, M. M. H. J., Vlenterie, R., IntHout, J., Engelen, L. J. L. P. G., Vrieling, A., & van de Belt, T. H. (2018). Most response-inducing strategies do not increase participation in observational studies: a systematic review and meta-analysis. Journal of Clinical Epidemiology, 99, 1–13. https://doi.org/10.1016/j.jclinepi.2018.02.019
